# Sustainable Synthesis of Bright Fluorescent Nitrogen-Doped Carbon Dots from *Terminalia chebula* for In Vitro Imaging

**DOI:** 10.3390/molecules27228085

**Published:** 2022-11-21

**Authors:** Raji Atchudan, Suguna Perumal, Thomas Nesakumar Jebakumar Immanuel Edison, Ashok K. Sundramoorthy, Sambasivam Sangaraju, Rajendran Suresh Babu, Yong Rok Lee

**Affiliations:** 1 School of Chemical Engineering, Yeungnam University, Gyeongsan 38541, Republic of Korea; 2 Department of Chemistry, Sejong University, Seoul 143747, Republic of Korea; 3 Department of Prosthodontics, Saveetha Dental College and Hospitals, Saveetha Institute of Medical and Technical Sciences, Poonamallee High Road, Velappanchavadi, Chennai 600077, Tamil Nadu, India; 4 National Water and Energy Center, United Arab Emirates University, Al Ain 15551, United Arab Emirates; 5 Laboratory of Experimental and Applied Physics, Centro Federal de Educação Tecnológica, Celso Suckow da Fonseca (CEFET/RJ), Av. Maracanã 229, Rio de Janeiro 20271-110, Brazil

**Keywords:** *Terminalia chebula*, carbon dot, fluorescence, cell viability, bioimaging

## Abstract

In this study, sustainable, low-cost, and environmentally friendly biomass (*Terminalia chebula*) was employed as a precursor for the formation of nitrogen-doped carbon dots (N-CDs). The hydrothermally assisted *Terminalia chebula* fruit-derived N-CDs (TC-CDs) emitted different bright fluorescent colors under various excitation wavelengths. The prepared TC-CDs showed a spherical morphology with a narrow size distribution and excellent water dispensability due to their abundant functionalities, such as oxygen- and nitrogen-bearing molecules on the surfaces of the TC-CDs. Additionally, these TC-CDs exhibited high photostability, good biocompatibility, very low toxicity, and excellent cell permeability against HCT-116 human colon carcinoma cells. The cell viability of HCT-116 human colon carcinoma cells in the presence of TC-CDs aqueous solution was calculated by MTT assay, and cell viability was higher than 95%, even at a higher concentration of 200 μg mL^−1^ after 24 h incubation time. Finally, the uptake of TC-CDs by HCT-116 human colon carcinoma cells displayed distinguished blue, green, and red colors during in vitro imaging when excited by three filters with different wavelengths under a laser scanning confocal microscope. Thus, TC-CDs could be used as a potential candidate for various biomedical applications. Moreover, the conversion of low-cost/waste natural biomass into products of value promotes the sustainable development of the economy and human society.

## 1. Introduction

In recent years, carbon-based nanomaterials have been attracting increasing attention from scientists due to their attractive physical and chemical properties, including their optical, electrical, and electronic properties [1,2,3]. Carbon dots are fluorescent nanoparticles with sizes in the range below 10 nm [4,5,6]. The fluorescent nature of carbon dots increases their applicability in a number of fields, including as sensors, and in fluorescence cell imaging and electrochemistry [7,8,9]. It is well documented that fluorescent carbon dots are better replacement materials for organic dyes and heavy-metal-based quantum dots, owing to their lower cytotoxicity, good water solubility, biocompatibility, and low photo-bleaching [10,11,12]. Furthermore, fluorescent carbon dots possess up-converting excitation-dependent emission properties. Therefore, the emission wavelength can be adjusted according to requirements, giving them good applicability in bio-imaging and flow cytometry. Generally, carbon dots can be synthesized using various methods, including hydrothermal carbonization, electrochemical exfoliation, solvothermal, and laser ablation techniques [13,14,15]. The hydrothermal carbonization of plant carbon sources is attracting a great deal of attention due to the simple, cost-effective, and easy experimental setup of this process. Plants/fruits are rich in secondary metabolites such as simple sugars, organic acids, tannins, and polyphenols, serving as good carbon sources for synthesizing carbon dots [16]. The formation of carbon dots mainly depends on the acidic, basic, and neutral constituents present in the plant source. Highly acidic phytoconstituents such as citric acid, tannic acid, tartaric acid, and neutral sugars yield good-quality carbon dots with high fluorescence quantum yield [17]. Many plant sources, such as *Prunus avium* fruit, *Phyllanthus acidus* fruit, *Prunus mume* fruit, *Chionanthus retusus* fruit, *Piper beetle* (Betel) leaf, etc., have been reported by our laboratory for the synthesis of carbon dots for use in sensors, cell imaging and electrochemical applications [18,19,20,21,22]. Generally, undoped carbon dots are less/non-fluorescent materials, whereas doping with electron-rich nitrogen, sulfur, and boron improves the fluorescent quantum yield due to the increase in surface defects. Moreover, the carbon precursor, doping agent, solvent, and size also influence the quantum yield of carbon dots [23].

The *Terminalia chebula* fruit belongs to the Combretaceae family and is native to India. This tree is widely available in India. *Terminalia chebula* fruit has excellent nutritional and medicinal value, and is applied directly in ancient Tamil Siddha medicinal systems. The main phytoconstituents of *Terminalia chebula* fruit are tannin, β-sitosterol, anthraquinones of about 20–40%, palmitic ester, oleic acid, linoleic acid, gallic acid, chebulinic acid, chebulagic acid, phenolic compounds, and polyphenols [24,25]. These phenolic compounds have antioxidant activities [25], and tannin and anthraquinone can be used to control the cathartic action [24]. Furthermore, the aqueous fruit extract of *Terminalia chebula* can be used as an anticaries agent, inhibiting the glycolysis of salivary bacteria [26]. As *Terminalia chebula* is widely available, and incorporates numerous constituents, including acids, flavonoids, and glycosides [27], it has been suggested that *Terminalia chebula* could act as an excellent carbon source for synthesizing carbon dots [28,29]. HCT-116 human colon carcinoma cells are an essential cell line that has been used extensively in therapeutic research and drug screening. HCT116 cells have been used in a variety of biomedical studies involving colon cancer proliferation and corresponding inhibitors. In order to understand the mechanism of cell growth, fluorescent dyes are important in tumorigenicity studies [30]. Therefore, this study describes the synthesis of nitrogen-doped carbon dots (N-CDs) using *Terminalia chebula* fruit extract by the hydrothermal carbonization method. Standard optical and surface tools are used to characterize the synthesized N-CDs. Finally, the *Terminalia chebula*-derived N-CDs are applied as fluorescent agents for cell imaging HCT-116 human colon carcinoma cells.

## 2. Results and Discussion

### 2.1. Structural Properties of Synthesized TC-CDs

**Figure 1:**
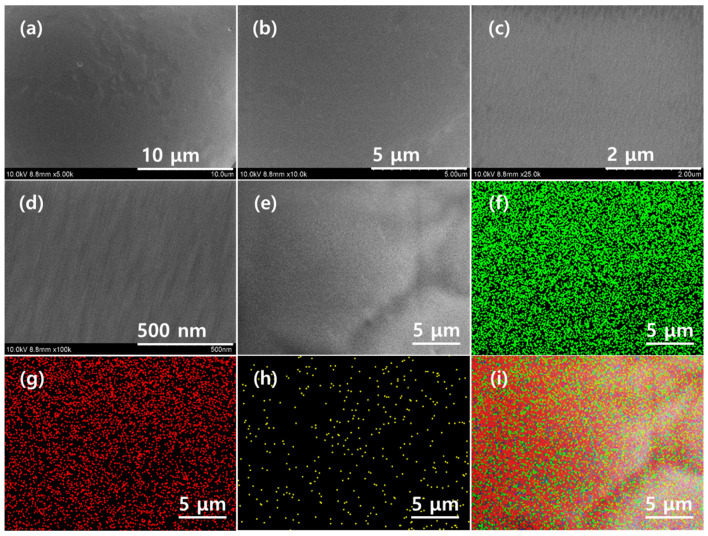
(**a**–**e**) FESEM images with different magnifications of the synthesized TC-CDs and the corresponding (**f**) carbon, (**g**) nitrogen, (**h**) oxygen, and (**i**) overlapping elemental maps of the presented elements.

The TC-CDs were successfully synthesized by a hydrothermally assisted carbonization route using sustainable, low-cost, and environmentally friendly biomass (Terminalia chebula) as a carbon source and water as a solvent. The surface morphology and elemental composition of the prepared TC-CDs were examined by field emission scanning electron microscopy with energy-dispersive X-ray spectroscopy (FESEM-EDS). As seen in the FESEM images (Figure 1a–e), carbon particles are formed into a fine surface because of the tiny size of the synthesized TC-CDs. This aggregation is due to the occurrence of a large number of functional groups. The hydrophilic surface functionalities form dangling bonds between the functional groups, resulting in a wrinkle-like surface morphology. The elemental composition of the samples from EDS mapping illustrates the uniform distribution of carbon (C), oxygen (O), and nitrogen (N) atoms in the synthesized TC-CDs (Figure 1f–i). Notably, heteroatoms, such as O and N atoms, are evenly distributed over the surface of the TC-CDs, confirming the successful incorporation of heteroatoms into the TC-CDs (Figure 1i). This result reveals that the surface of the synthesized TC-CDs is enriched with oxygen- and nitrogen-containing functional groups.

**Figure 2:**
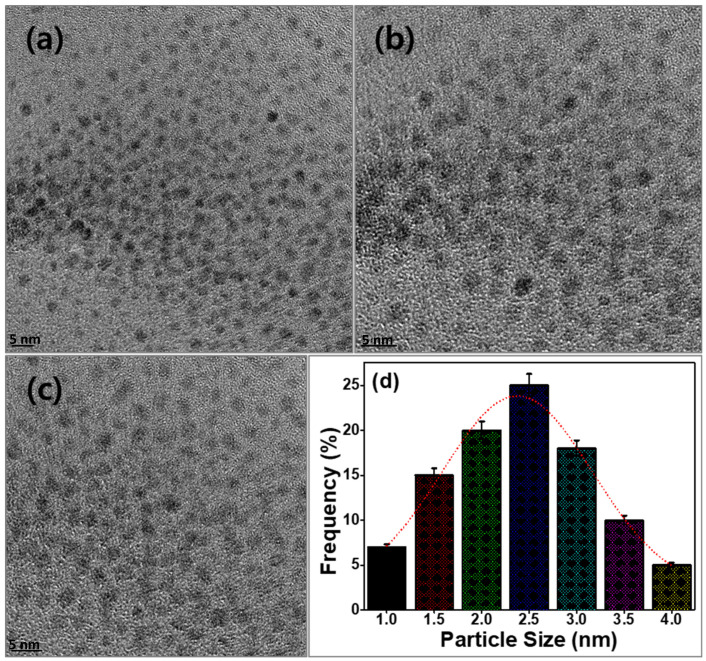
(**a**–**c**) HRTEM images with different magnifications and (**d**) the corresponding particle-size histogram of the synthesized TC-CDs.

Furthermore, the morphology and crystallinity/graphitic nature of the synthesized TC-CDs were determined by analysis using high-resolution transmission electron microscopy (HRTEM). It can be observed from Figure 2a–c that the synthesized TC-CDs had a nearly spherical shape and uniform dispersity. Additionally, the HRTEM images demonstrate that the TC-CDs possessed a moderately crystalline/graphitic nature in the inner core, with a lattice spacing of ~0.21 nm, which is consistent with the (100) faces of graphitic sheets, while the outer shell of the spherical TC-CDs had a nearly amorphous nature [31]. The amorphous structure of the outer shell of the spherical TC-CDs might be due to the presence of functional groups on the surface of the TC-CDs. The particle size distribution range between 1 nm and 4 nm, with an average size of around 2.5 nm, was obtained from the particle-size histogram of the synthesized TC-CDs (Figure 2d).

**Figure 3:**
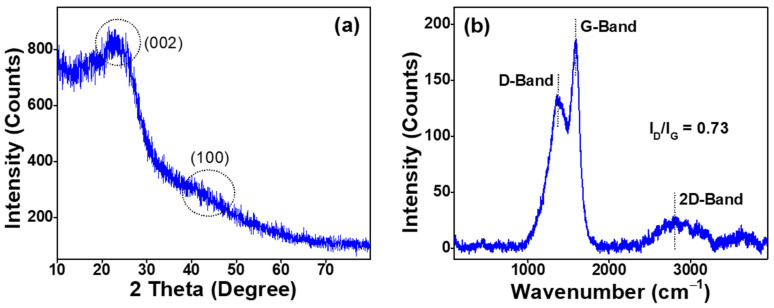
(**a**) XRD pattern and (**b**) Raman spectrum of the synthesized TC-CDs.

X-ray Powder Diffraction (XRD) patterns revealed the crystalline/graphitic character of the synthesized TC-CDs. In the XRD spectrum (Figure 3a), a broad diffraction peak centered at 24° corresponded to the (002) plane, a plane that is responsible for typical graphene. The corresponding interplanar space was calculated as being 0.37 nm, which is higher than the regular d-spacing of graphene/graphite. The increment in interplanar spacing suggests that the synthesized TC-CDs possessed moderate graphitic characteristics, with considerable amorphous behavior [32]. This amorphous characteristic might be a result of the damaged/disordered outer part and or the occupied functional groups on the surface/edges of the synthesized TC-CDs. Additionally, the noisier broad peak might be attributed to the very smaller size of the carbon particles or the disordered carbon atoms and amorphous graphitic structure [33,34]. The second minor peak at around 42.5° was ascribed to the (100) plane of standard graphene, and the corresponding interplanar spacing was calculated to be nearly 0.21 nm. This result for interlayer distance coincides nicely with the HRTEM results. Furthermore, the Raman spectrum revealed the crystalline/graphitic character of the TC-CDs. The Raman spectrum of the synthesized TC-CDs (Figure 3b) presented two broad absorption bands without clear separation at around 1360 cm^−1^, which was attributed to the D-band (sp^3^ hybrid carbon), hybrid vibration (A_1g_) mode of the disordered edges associated with amorphous carbon in the TC-CDs, and at 1590 cm^−1^, which was ascribed to the G band (sp^2^ hybrid carbon), in-plane vibration E_2g_ mode of the graphitic domains associated with crystalline carbon core in the TC-CDs [35,36,37,38]. The coexistence of the two bands indicated that the TC-CDs had a graphitic structure with a disordered cross-section. The intensity ratio of the Raman D-band and G-band is commonly used to determine the quality of graphene sheets or carbon nanomaterials/carbon dots [39,40]. The D-band and G-band (I_D_/I_G_) intensity ratio of the TC-CDs was 0.73, characterizing the permissible degree of order in the core and the percentage of sp^3^/sp^2^ carbon atoms in the TC-CDs. Compared to the G-band of the TC-CDs, the broadening of the D-band suggests smaller graphitic domain sizes, leading to smaller distributions. A broad 2D-band at around 2800 cm^−1^ indicates the double resonance of the D-band. The shape and position of the 2D-band provide information about the number of layers in the graphene sheet [41]. The small size and low intensity of the 2D-band suggests that the synthesized TC-CDs are probably smaller in size and thinner in structure. Notably, the bands (D and G) have unclear separations in this spectrum. Therefore, it is a little difficult to judge the degree of order (degree of graphitization/crystallinity) on the basis of the intensity ratio of the D-band and the G-band of the synthesized TC-CDs. Thus, the degree of graphitization/crystallinity was further demonstrated using the area ratio of the D-band and the G-band of the synthesized TC-CDs. The D-band and G-band were deconvoluted with the same width to quantify the D-area (A_D_) and G-area (A_G_), as shown in Supplementary Materials: Figure S1. The calculated ratio of A_D_/A_G_ was around 0.81, indicating a graphitic carbon core with disordered domains associated with the smaller amount of amorphous carbon on the edges of the TC-CDs. These results strongly suggest that the synthesized TC-CDs exhibited a satisfactory degree of order.

**Figure 4:**
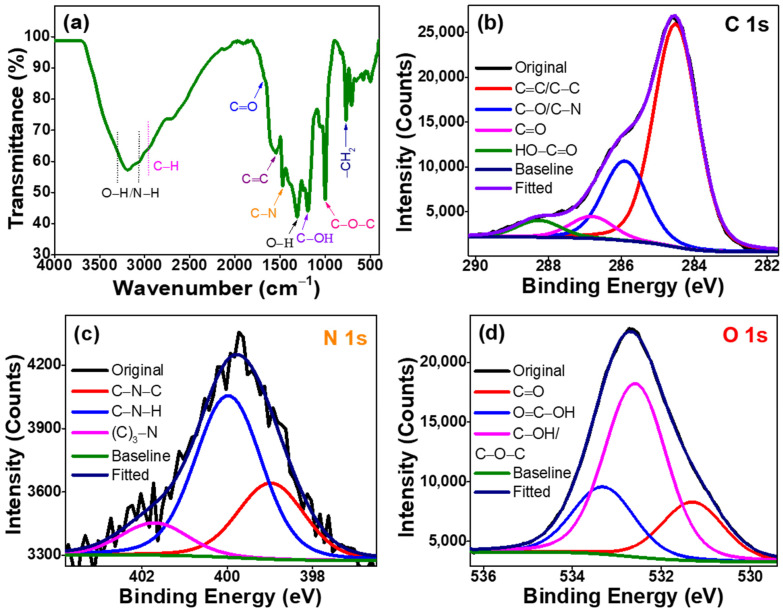
(**a**) ATR-FTIR spectrum of synthesized TC-CDs. XPS spectra of synthesized TC-CDs; (**b**) high-resolution spectrum of C 1s, (**c**) high-resolution spectrum of N 1s, and (**d**) high-resolution spectrum of O 1s levels with deconvolution.

The surface functional groups and chemical composition of the synthesized TC-CDs were investigated using the attenuated total reflection Fourier-transform infrared (ATR-FTIR) spectroscopy technique (Figure 4a). The broad absorption band centered around 3400–3000 cm^−1^ can be attributed to the characteristic stretching vibrations of the O–H/N–H groups, which originated from the natural biomass and or physically adsorbed water molecules [42,43]. The FTIR spectrum showed weak shoulder peaks at around 2958 and 1698 cm^−1^, affirming the existence of C–H symmetric/asymmetric and carboxyl/carbonyl (C=O) stretching vibrations, respectively [44,45,46]. The strong and distinct absorption signals at 1565, 1475, 1315, and 1200 cm^−1^ were attributed to the presence of the C=C stretching, C–N stretching, O–H/N–H bending, and C–OH stretching vibrations, respectively [47,48]. The absorption signal at 1005 cm^−1^ corresponds to the C–O–C stretching mode in the carbon framework [49]. The sharp absorption bands at 767 cm^−1^ can be attributed to teh out-plane aromatic –CH_2_ bending vibration on the surface of the TC-CDs [50]. The presence of a C=C peak indicates that the TC-CDs are made of graphitic structure, whereas the O–H, C=O, and C–H vibration peaks suggest that the surfaces of TC-CDs were fully covered by the presence of hydroxyl, carbonyl, and amine moieties [51]. These hydrophilic functional groups are probably responsible for the excellent dispersibility of TC-CDs in water.

To further reveal the elemental composition, elemental state, and functional moieties on the surface of the TC-CDs, X-ray photoelectron spectroscopy (XPS) studies were performed. As shown in Figure S2, the synthesized TC-CDs were primarily composed of C, N, and O, with atomic percentages of 70, 5, and 25, respectively. The absence of other residual elements highlighted the purity of the synthesized TC-CDs. The high-resolution XPS spectrum of C 1s could be deconvoluted into four prominent peaks at 284.5, 285.9, 286.8, and 288.3 eV, corresponding to sp^3^/sp^2^ carbons (C–C/C=C), C–N/C–O, C=O, and HO–C=O, respectively [52,53,54]. The characteristic peak of C=C indicates that distinctive graphite structures might be presented in the synthesized TC-CDs. The deconvolution of the nitrogen peak is displayed in Figure 4c, and the high-resolution spectrum for N 1s exhibits three significant peaks at 399.0, 400.0, and 401.8 eV, which can be attributed to C–N–C, C–N–H, and (C)_3_–N, respectively [55]. The high-resolution XPS spectrum for the O 1s level shows three peaks at 531.3, 532.6, and 533.3 eV, which can be attributed to the C=O, C–OH/C–O–C, and HO–C=O functional groups, respectively [56,57]. One-fourth of oxygen carbon atoms were functionalized with carbon, indicating that the TC-CDs have a large number of oxygen moieties. The XPS and FTIR results showed that the TC-CDs were highly functionalized with oxygen- and nitrogen-containing moieties. This could be the main reason for which the TC-CDs possess excellent water dispersibility and long-term colloidal stability.

### 2.2. Optical Properties of Synthesized TC-CDs

**Figure 5:**
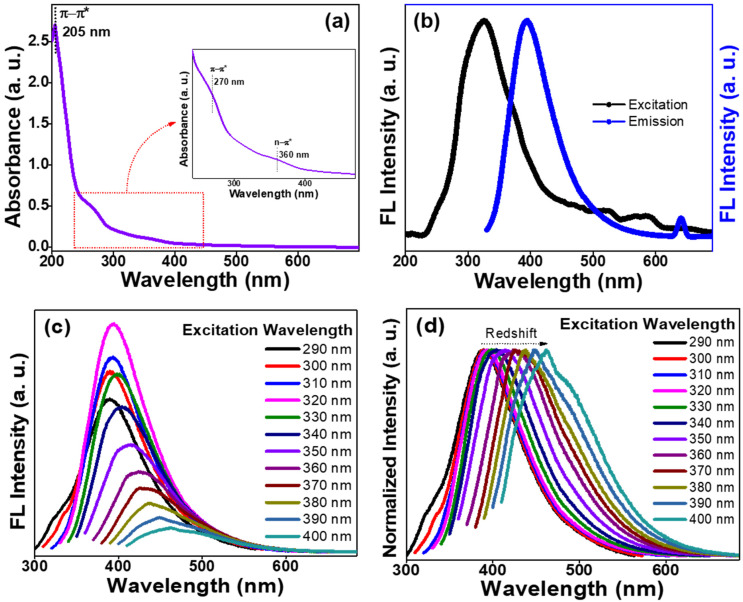
(**a**) UV–vis absorption spectrum of synthesized TC-CDs; (**b**) fluorescence excitation and emission spectra of the synthesized TC-CDs; (**c**) fluorescence emission spectra of the synthesized TC-CDs were measured with 10 nm increments of excitation wavelength in the range from 290 to 400 nm, and (**d**) the corresponding normalized fluorescence emission spectra.

**Figure 6:**
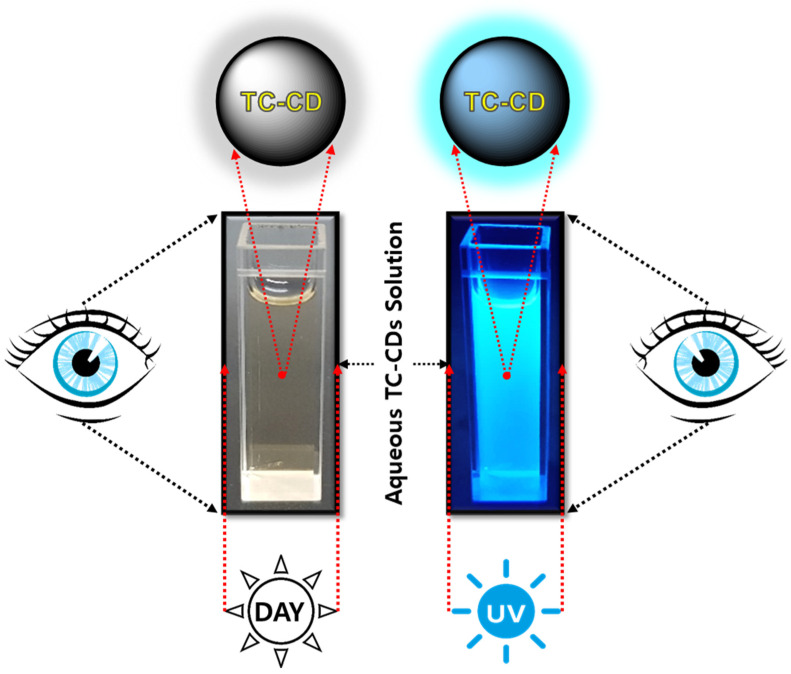
Photographic images of aqueous TC-CDs solutions exposed under daylight (left) and 365 nm UV light (right) as viewed by the naked eye.

To examine the optical properties of the synthesized TC-CDs, the ultraviolet–visible (UV–vis) absorption and fluorescence spectra were recorded in aqueous solution. The absorption spectrum of the TC-CDs (Figure 5a) displays three peaks, a narrow peak around 205 nm and a broad shoulder hump around 270 and 360 nm, which could be associated with the π–π* transition (C=C) and n–π* transition (C=O) of the nanocarbon, respectively [58,59,60]. Figure 5b displays the fluorescence excitation and emission spectra of the synthesized TC-CDs in water. It can be seen from the spectra that the maximum fluorescence excitation and emission were observed at 320 and 395 nm, respectively, which indicates that the maximum fluorescence emission occurred at 395 nm upon excitation at 320 nm. The full width at half maximum of the fluorescence emission spectrum was around 81 nm, which confirms the narrow size distribution of the synthesized TC-CDs [58]. To examine the excitation-dependent emission properties, the fluorescence emissions were recorded at different excitation wavelengths from 290 to 400 nm, as illustrated in Figure 5c. The fluorescence emission intensity increased progressively when the excitation wavelength was increased from 290 to 320 nm, while further increasing the excitation wavelength to 400 nm caused a decline in the fluorescence intensity. The fluorescence spectra of the synthesized TC-CDs clearly display excitation-wavelength-dependent fluorescence emissions upon changing the excitation wavelength, which is more common in carbon dots due to the presence of surface defects and different functional groups [18,61,62]. In addition, it can be seen that the fluorescence emission peak shifted to higher wavelengths (redshift) with increasing excitation wavelengths. The redshift in the fluorescence emission can be clearly observed in Figure 5d. These fluorescence emission properties, which are tunable by excitation wavelength, represent valuable characteristics of TC-CDs, making them applicable as fluorescent nanoprobes for multicolor imaging. The fluorescence quantum yield of the TC-CDs at an excitation wavelength of 320 nm was calculated to be 15%, using quinine sulphate as a reference, and this quantum yield is comparable to carbon dots originating from other biomass (natural resources). The reasonable quantum yield obtained might be due to the presence of a high degree of oxygen- and nitrogen-containing functional groups on the surface of the TC-CDs. To support the fluorescence emission of synthesized TC-CDs, the TC-CDs solution in water was exposed under daylight (normal light) and UV light (365 nm) (Figure 6). The TC-CDs aqueous solution appeared transparent and pale yellow under daylight. However, the TC-CDs aqueous solution emitted a bright cyan-blue color (fluorescence) under UV light that could be easily observed with the naked eye.

Furthermore, their photostability is well known, and this photostability of the synthesized TC-CDs is intrinsic to their real-time (practical) applications. Hence, the TC-CDs aqueous solution was continuously exposed to UV light illumination for 120 min, and the stability (fluorescence intensity) of the TC-CDs was investigated. The fluorescence intensity of the TC-CDs remained almost the same, even after continuous UV light irradiation for 120 min (Figure S3). The digital photographs of the TC-CDs aqueous solution under 365 nm UV light before and after UV light irradiation provide further support for the photostability of the synthesized TC-CDs. In the photographic image (inset Figure S3), insignificant changes can be observed by the naked eye under continuous exposure to UV light excitation for 120 min. These results confirm that the TC-CDs have anti-photobleaching (excellent photostability) properties, making them promising fluorescence candidates for practical applications [44,63].

### 2.3. Bioimaging Applications of Synthesized TC-CDs

The synthesized TC-CDs were used directly as a staining agent (fluorescent nanoprobe) for cellular imaging due to their ideal properties, such as tunable fluorescence emission, reasonable quantum yield, excellent water dispersibility, durable anti-photobleaching, low cytotoxicity, and good biocompatibility. A cell viability test was conducted before performing cellular imaging because the cell viability assay was essential for cellular imaging [64]. Hence, the cytotoxicity of the TC-CDs against human colon carcinoma cells (HCT-116) was examined using the 3-(4,5-dimethylthiazol-2-yl)-2,5-diphenyl tetrazolium bromide (MTT) assay. The resulting bar graph of the cytotoxicity of TC-CDs at different concentrations (0–200 μg mL^−1^) against HCT-116 cells is presented in Figure S4. These results demonstrated that the synthesized TC-CDs exhibited insignificant cytotoxicity towards HCT-116 cells, even at a high concentration of 200 μg mL^−1^. The cell viability was greater than 95%, even after 24 h incubation. The outstanding viability of HCT-116 cells in the presence of the TC-CDs confirmed that the synthesized TC-CDs exhibit excellent good biocompatibility with HCT-116 cells. Thus, the synthesized TC-CDs were used directly as a fluorescent nanoprobe for the imaging of HCT-116 cells.

**Figure 7:**
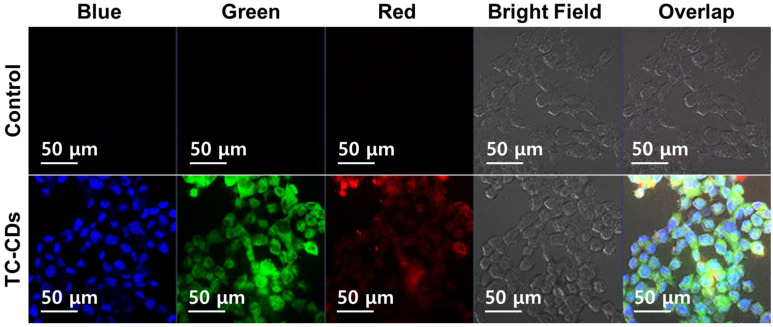
Confocal fluorescence images of human colon cancer (HCT-116) cells in the presence and absence of TC-CDs incubated at 24 h using different filters with excitation wavelengths of 405 (blue), 488 (green), and 555 nm (red) and in a bright field. Concentration of TC-CDs is 100 μg mL^−1^.

Figure 7 presents the confocal microscopy images of HCT-116 cells with and without the conjugation of TC-CDs after 24 h incubation. Various filters, such as blue, green, and red, with wavelengths of 405, 488, and 555 nm, respectively, were used for the imaging of the HCT-116 cells. Figure 7 demonstrates that the HCT-116 cells did not show any emission signal without the conjugation of TC-CDs under different excitation wavelengths. TC-CDs with the conjugation of HCT-116 cells displayed bright emissions in blue, green, and red colors when varying the filter wavelengths to 405, 488, and 555 nm, respectively. The bright multicolor emission of HCT-116 cells is due to the excitation-dependent emission of synthesized TC-CDs. The overlapped image displays multiple colors, also revealing that the TC-CDs have fluorescence behavior that is tunable by varying the excitation wavelengths. TC-CDs are easily internalized and homogenously distributed in the entire body of the HTC-116 cells because of the smaller size of the TC-CDs. Furthermore, this quick internalization and homogenous distribution might be due to the hydrophilic nature of the TC-CDs; the hydrophilicity is because of the hydroxyl, carbonyl, carboxylic acid, and amine/amide groups on the surface of TC-CDs. Ultimately, these results revealed that the synthesized TC-CDs have potential as fluorescent nanoprobes for the imaging of HTC-116 cells [65,66]. Additionally, these results demonstrate that the synthesized TC-CDs can act as nanocarriers for cell labeling and drug delivery.

## 3. Conclusions

In summary, novel and highly efficient TC-CDs were successfully synthesized from *Terminalia chebula* fruit via an eco-friendly one-step hydrothermal method. The resulting TC-CDs had a uniform particle size distribution with an average diameter of 2.5 nm. Various combinations of nitrogen and oxygen moieties were present on the surfaces of the TC-CDs, leading to their having enhanced optical properties. The prepared TC-CDs were highly dispersible in water because of their high degree of hydrophilicity. The as-synthesized TC-CDs possessed a redshifted excitation-dependent emission behavior, with intensity gradually decreasing with increases in excitation beyond 320 nm. The TC-CDs showed durable photostability. Additionally, the TC-CDs exhibited extremely low cytotoxicity, even at high concentrations (200 μg mL^−1^), and good biocompatibility against human colon carcinoma cells (HCT-116), as verified by MTT assay. The synthesized TC-CDs were used as a staining agent for cell imaging as a result of their small size, multicolor emissions, high water dispersibility, low cytotoxicity, and good biocompatibility. The TC-CDs were efficiently internalized into the HCT-116 cells and emitted multicolor fluorescence, demonstrating tremendous potential applications in the fields of bioimaging and biolabeling. Moreover, this work offers a new platform for multicolor cell imaging and a biocompatible fluorescent probe to replace harmful organic dyes, while additionally transforming cheap biomass into a high value-added product through the conversion of waste biomass.

## 4. Materials and Methods

### 4.1. Materials

*Terminalia Chebula* fruits were collected from Tamil Nadu, India. Aqueous ammonia (NH_4_OH, 25%) was purchased from Sigma-Aldrich, Republic of Korea. Phosphate-buffered saline (PBS), N-(2-hydroxyethyl)piperazine-N’-(2-ethane sulfonic acid) (HEPES), p-formaldehyde, quinine sulfate, and dimethyl sulfoxide (DMSO) were purchased from Sigma-Aldrich, Seoul, Republic of Korea. 3-(4,5-dimethylthiazol-2-yl)-2,5-diphenyltetrazolium bromide (MTT) was purchased from Generay Biotech, Shanghai, China. HCT-116 human colon cancer cells were purchased from ATCC, CCL-247, Manassas, VA, USA. All the chemicals were used as purchased, and distilled water was used throughout this study.

### 4.2. Synthesis of TC-CDs

**Scheme 1:**
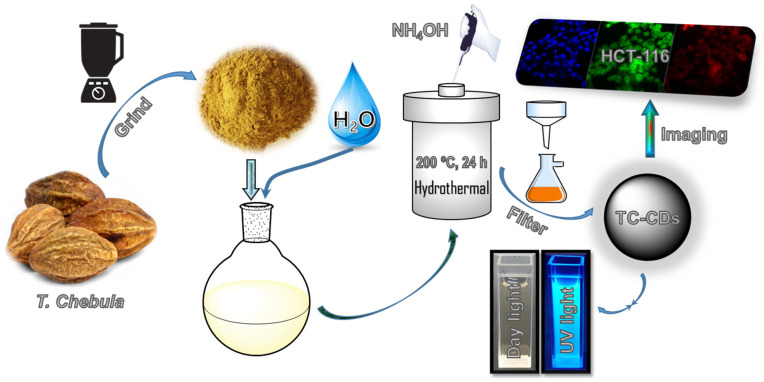
The detailed preparation and applications of TC-CDs from *Terminalia chebula* fruit.

Fluorescent TC-CDs were synthesized from *Terminalia chebula* fruit using an economical one-step hydrothermal route (Scheme 1). In a typical synthesis, 500 mg of *Terminalia chebula* powder was mixed with 50 mL of distilled water and stirred well. The solution of pH was adjusted from 3.5 to 7.0. Subsequently, the mixture was transferred into a Teflon-lined stainless steel autoclave and heated at 200 °C for 24 h. After completion of hydrothermal treatment, the autoclave was cooled down to room temperature. The resulting brownish-yellow solution was separated through a mixed cellulose ester membrane filter (pore size 0.22 μm). Afterward, the resulting brownish-yellow solution was freeze-dried to obtain the TC-CDs. The final TC-CDs were used for further analysis.

## Data Availability

Not applicable.

## Supplementary Materials

The following supporting information can be downloaded at: https://www.mdpi.com/article/10.3390/molecules27228085/s1. Instrumentation methods, quantum yield measurement, photobleaching measurements, cell culture, cell viability assay, and microscopy analysis of the synthesized TC-CDs. Figure S1: (a) Raman deconvoluted the spectrum of synthesized TC-CDs and (b) the corresponding magnified view; Figure S2: XPS survey scan spectrum of synthesized TC-CDs; Figure S3: Fluorescence spectrum of synthesized TC-CDs under different irradiation times (365 nm UV light) 0 min (before) and 120 min (after); Figure S4. Bar graph illustrating the cell viability (%) using the MTT assay of HTC-116 after 24 h of incubation with synthesized TC-CDs.

## Sample Availability

Samples of the compounds are available from the authors on request.
