# Supplementary material for: Sustainable Synthesis of Bright Fluorescent Nitrogen-Doped Carbon Dots from Terminalia chebula for In Vitro Imaging
Source: Molecules. 2022 Nov 21;27(22):8085. doi: 10.3390/molecules27228085 (PMC9693165; doi:10.3390/molecules27228085)
Supplement: Supplementary file 1 [file molecules-27-08085-s001.zip › molecules-2033379-supplementary.pdf]

molecules

MDPI

Supplementary Materials

Sustainable Synthesis of Bright Fluorescent Nitrogen-Doped Carbon Dots from *Terminalia Chebula* for *In Vitro* Imaging

Raji Atchudan [1,*,†], Suguna Perumal [2,†], Thomas Nesakumar Jebakumar Immanuel Edison [1,†], Ashok K. Sundramoorthy [3,†], Sambasivam Sangaraju [4], Rajendran Suresh Babu [5,†] and Yong Rok Lee [1,*]

1 School of Chemical Engineering, Yeungnam University, Gyeongsan 38541, Republic of Korea
2 Department of Chemistry, Sejong University, Seoul 143747, Republic of Korea
3 Department of Prosthodontics, Saveetha Dental College and Hospitals, Saveetha Institute of Medical and Technical Sciences, Poonamallee High Road, Velappanchavadi, Chennai 600077, Tamil Nadu, India
4 National Water and Energy Center, United Arab Emirates University, Al Ain 15551, United Arab Emirates
5 Laboratory of Experimental and Applied Physics, Centro Federal de Educação Tecnológica, Celso Suckow da Fonseca (CEFET/RJ), Av. Maracanã 229, Rio de Janeiro 20271-110, Brazil
* Correspondence: atchudanr@yu.ac.kr (R.A.); yrlee@yu.ac.kr (Y.R.L.)
† These authors contributed equally to this work.

Citation: Atchudan, R.; Perumal, S.; Edison, T.N.J.I.; Sundramoorthy, A.K.; Sambasivam, S.; Babu, R.S.; Lee, Y.R. Sustainable Synthesis of Bright Fluorescent Nitrogen-Doped Carbon Dots from *Terminalia Chebula* for In Vitro Imaging. *Molecules* **2022**, *27*, 8085. https://doi.org/10.3390/molecules27228085

Academic Editors: Pengxiang Hou and Maoshuai He

Received: 31 October 2022
Accepted: 18 November 2022
Published: 21 November 2022

Publisher's Note: MDPI stays neutral with regard to jurisdictional claims in published maps and institutional affiliations.

Copyright: © 2022 by the authors. Licensee MDPI, Basel, Switzerland. This article is an open access article distributed under the terms and conditions of the Creative Commons Attribution (CC BY) license (https://creativecommons.org/licenses/by/4.0/).

Instrumentation Methods

Terminalia chebula fruit-derived nitrogen-doped carbon dots (TC-CDs) were characterized by various physicochemical analytical techniques such as field emission scanning electron microscopy (FESEM) with energy-dispersive X-ray spectroscopy (EDS), high-resolution transmittance electron microscopy (HRTEM), X-ray diffraction (XRD), Raman spectroscopy, attenuated total reflectance Fourier-transform infrared (ATR-FTIR) spectroscopy, X-ray photoelectron spectroscopy (XPS), Ultraviolet-visible (UV-vis) absorption spectroscopy, and fluorescence spectroscopy. FESEM with EDS analysis was carried out on a Hitachi S-4800 equipped with EDX at an accelerating voltage of 10/15 kV. TEM/HRTEM images were performed with a JEOL JEM transmission electron microscope with an operating accelerating voltage of 120 kV. XRD measurements were carried out using a PANalytical X'Pert3 MRD diffractometer with monochromatized Cu Kα radiation (λ = 1.54 Å) at 40 kV and 30 mA and were recorded in the range from 10 to 80° (2θ). Raman spectrum was recorded on XploRA Micro-Raman spectrophotometer (Horiba) with the ranges between 50 and 4000 cm^{-1} at the core research support center for natural products and medical materials of Yeungnam University. ATR-FTIR spectra were recorded in transmittance mode on a Perkin Elmer Spectrum Two in the wavenumber range from 400 to 4000 cm^{-1} by the addition of 8 scans at a resolution of 8 cm^{-1}. XPS spectra were achieved using a K-Alpha (Thermo Scientific). CasaXPS software was used for the deconvolution of the high-resolution XPS spectra. UV-vis absorption spectra were recorded from 200 to 700 nm using an OPTIZEN 3220UV spectrophotometer. Excitation and emission fluorescence spectra were recorded using a Hitachi F-7000 fluorescence spectrophotometer using a 1 cm^3 path-length quartz cell. The excitation wavelength was varied to determine the maximum emission intensity. The slit width was fixed at 5 nm, and the scan speed was set to 400 nm/min. Cellular imaging was performed using a confocal laser scanning microscope (X400; LSM700, Carl Zeiss, Oberkochen, Germany).

Quantum Yield Measurement of Synthesized TC-CDs

The quantum yield (QY) of the synthesized TC-CDs was calculated by using quinine sulfate in 0.1 M H_2SO_4 (QYR is 0.54) as a standard reference and was calculated using the following equation (1):

$$QY = QY_R \frac{I_S A_R (n_S)^2}{I_R A_S (n_R)^2} \quad (1)$$

where, "I" is the measured integrated fluorescent emission intensity, "n" is the refractive index of the solvent, and "A" is the absorbance (intensity). The subscript "R" and "S" refer to the known fluorescent reference and standard for the synthesized sample, respectively.

Photobleaching Measurements of Synthesized TC-CDs

The photostability (photobleaching) of the synthesized TC-CDs was examined by continuous irradiation under UV light (365 nm) for 120 min. The fluorescence intensity of the TC-CDs aqueous solution was measured before and after UV-light irradiation.

Cell Culture, Cell Viability Assay, and Microscopy Analysis of Synthesized TC-CDs

HCT-116 was cultured in Roswell Park Memorial Institute (RPMI) 1640 medium containing 25 mM HEPES buffer, 10% heat-inactivated fetal bovine serum, and 1% penicillin/streptomycin under 5% CO_2 atmosphere at 37 °C until coming to a composition, subsequently treated with different concentrations of M-CDs (0–200 μg mL^{-1}) for 24 h. MTT (3-(4, 5-dimethylthiazol-2-yl)-2,5-diphenyl tetrazolium bromide) cell viability test was performed to determine the cytotoxicity of the synthesized TC-CDs. The wells in the microtiter plate were incubated for 4 h at 37 °C in the dark in a humidified chamber with the addition of MTT solution (5 mg MTT mL^{-1} in phosphate-buffered saline (PBS)). Finally, insoluble purple-formazan crystals were observed in TC-CDs treated cells, which were subsequently dissolved in dimethyl sulfoxide (DMSO), and optical density was measured at 540 nm.

Cells were cultured on coverslips in 6-well plates for fluorescent confocal imaging. At confluence, staining was achieved by incubating the cells in the presence and absence of the TC-CDs in a humidified chamber at 37 °C for 24 h. The staining of cells was fixed with 4% p-formaldehyde, washed with PBS 3 times, and then mounted in a fluorescence mounting medium (Dako North America Inc., Carpinteria, CA, USA). Then the fluorescence of the stained cells was imaged with appropriate filter sets (blue (405 nm), green (488 nm), and red (555 nm)) as well as with bright field (BF) on confocal microscopy (X400; LSM700, Carl Zeiss, Oberkochen, Germany).

Structural Properties of Synthesized TC-CDs

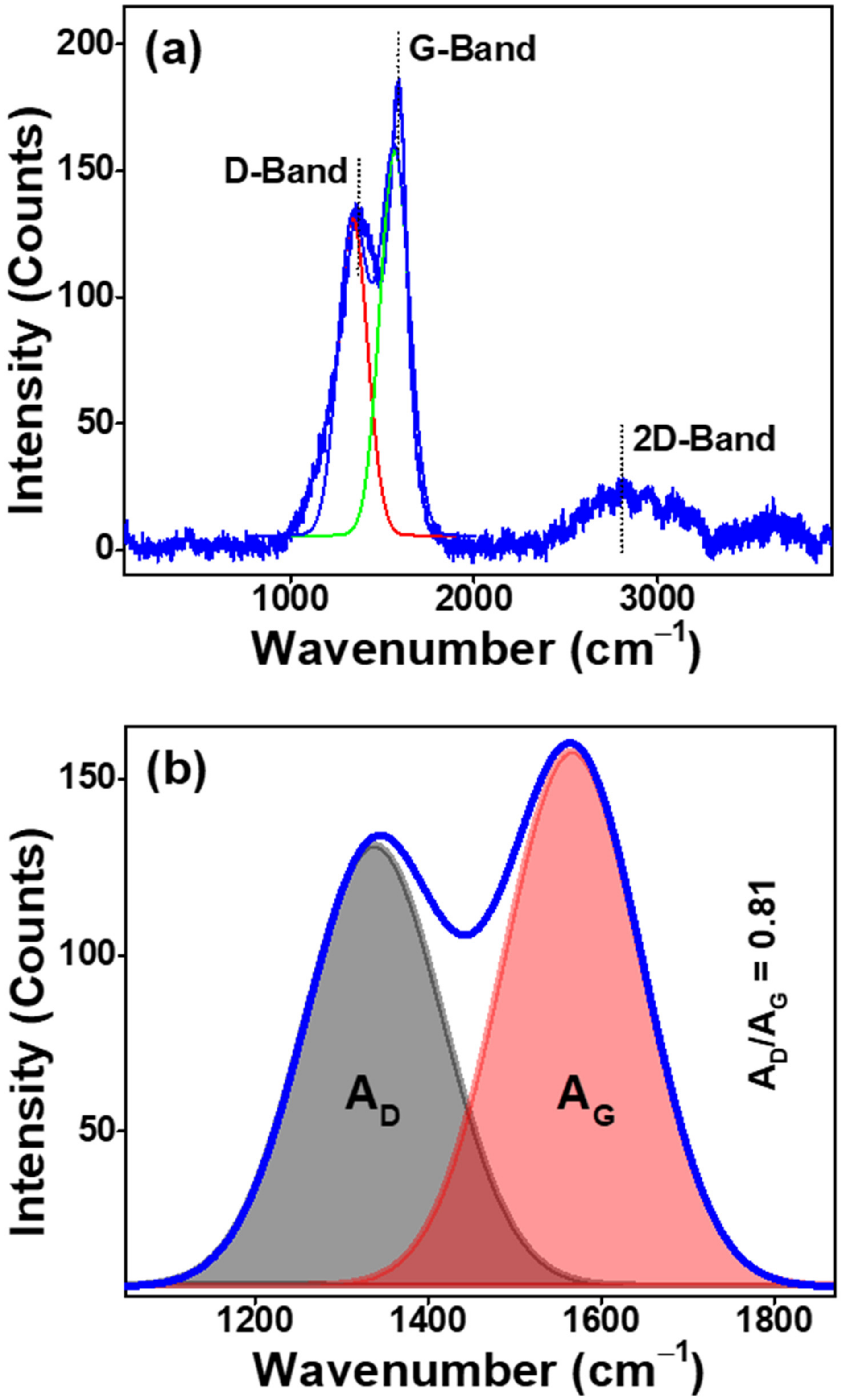

Figure S1. (a) Raman deconvoluted the spectrum of synthesized TC-CDs and (b) the corresponding magnified view.

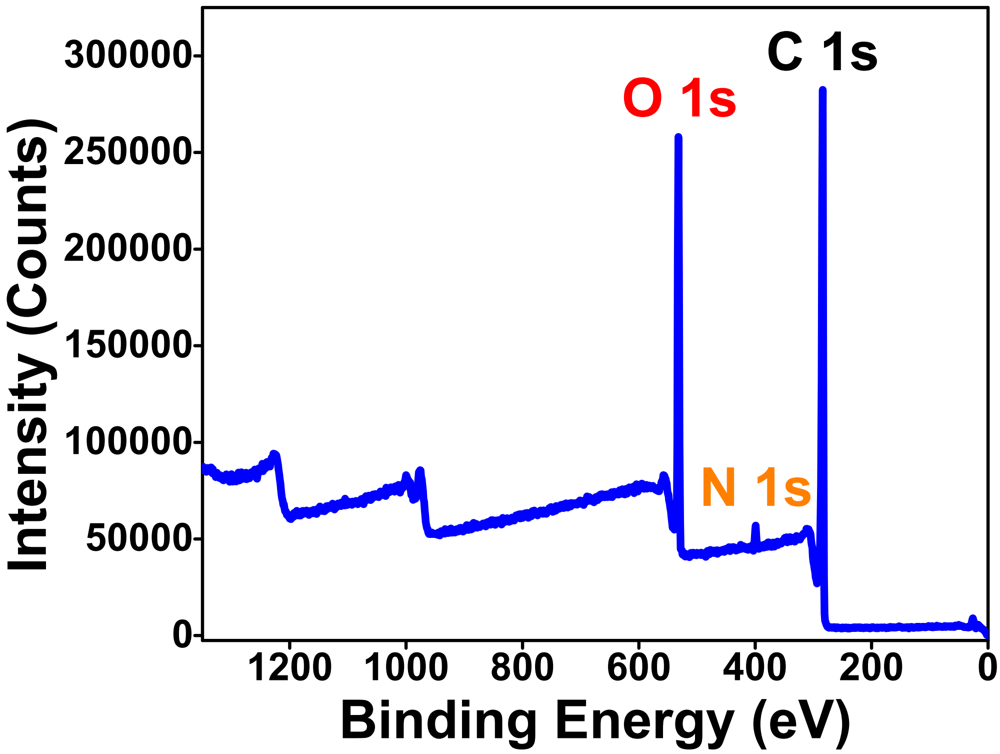

Figure S2. XPS survey scan spectrum of synthesized TC-CDs.

Optical Properties of Synthesized TC-CDs

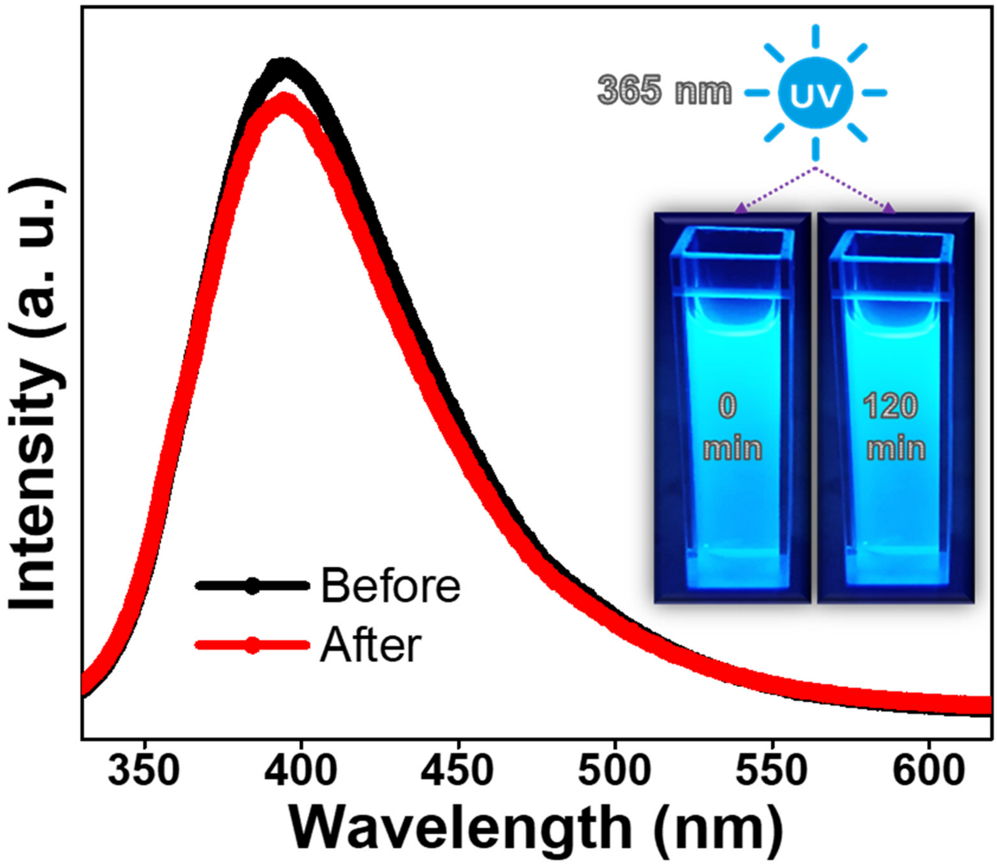

Figure S3. Fluorescence spectrum of synthesized TC-CDs under different irradiation times (365 nm UV light) 0 min (before) and 120 min (after).

Cytotoxicity Result of Synthesized TC-CDs

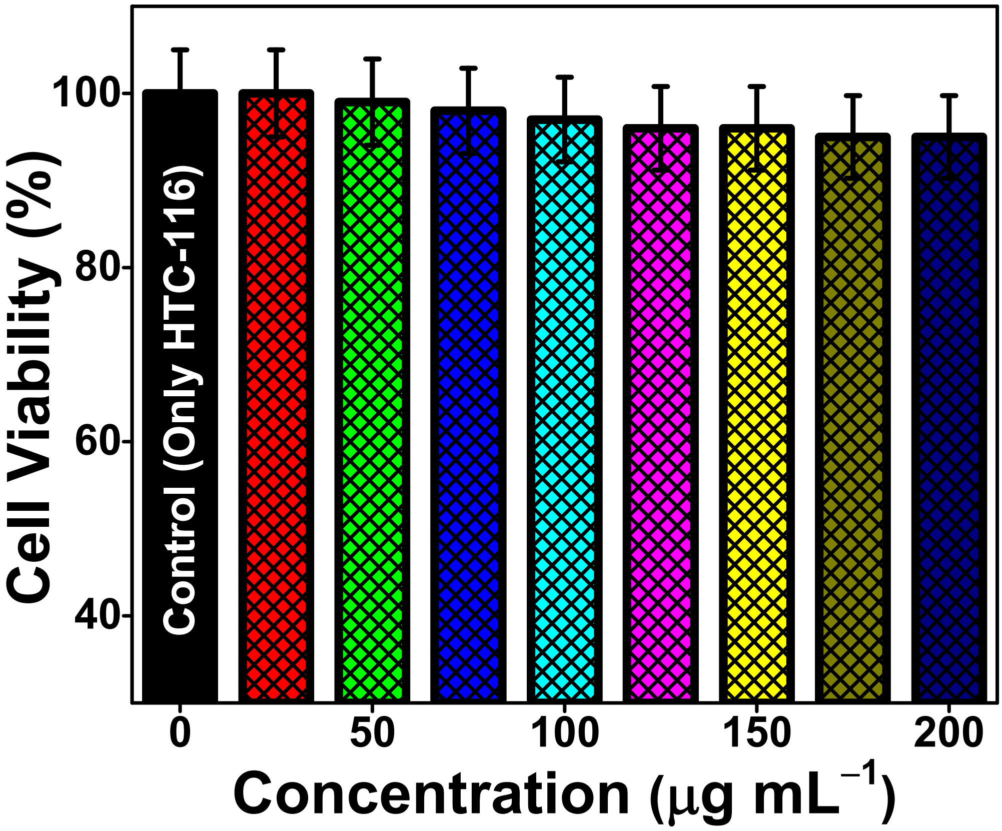

Figure S4. Bar graph illustrating the cell viability (%) using the MTT assay of HTC-116 after 24 h of incubation with synthesized TC-CDs.
